# Awareness, knowledge and self-reported test rates regarding Hepatitis B in Turkish-Dutch: a survey

**DOI:** 10.1186/1471-2458-10-512

**Published:** 2010-08-24

**Authors:** Ytje JJ van der Veen, Hélène ACM Voeten, Onno de Zwart, Jan Hendrik Richardus

**Affiliations:** 1 Erasmus MC, University Medical Center Rotterdam, Dept. of Public Health, Rotterdam, the Netherlands; 2 Municipal Public Health Service GGD Rotterdam-Rijnmond, Rotterdam, the Netherlands

## Abstract

**Background:**

Hepatitis B virus infection is an important health problem in the Turkish community in the Netherlands. To prevent transmission and progression of the disease in this community, increased screening is necessary. This study aimed to determine 1) the levels of awareness and knowledge regarding hepatitis B, comparing these in tested and non-tested Turkish-Dutch in Rotterdam; 2) the self-reported hepatitis B test status in this population, and how this is related to demographic characteristics, knowledge and awareness.

**Methods:**

We conducted a postal survey amongst first and second generation migrants, aged 16 - 40 years.

**Results:**

The response rate was 30.2% (n = 355 respondents). Levels of awareness and knowledge regarding hepatitis B were low, as the majority of respondents (73%) never thought about the disease and 58% of the respondents scored 5 or less out of ten knowledge items. Weighted analysis of self-reports showed a test rate of 15%, and a vaccination rate of 3%. Regression analysis showed that having been tested for hepatitis B was related to being married and higher levels of awareness and knowledge.

**Conclusions:**

This study shows low levels of hepatitis B awareness and knowledge in the Turkish community in Rotterdam. Self-reported test rates are lower in people who are not currently married, and in those who have low levels of awareness and knowledge. Especially, knowledge about the consequences of hepatitis B, such as liver cancer, was lacking. Therefore, a health promotion intervention should foremost raise awareness, and increase knowledge on the seriousness of this disease.

## Background

Hepatitis B virus (HBV) infection is one of the major infectious diseases in the world [1]. The endemic status of the Netherlands is classed as low, but population migration from high or medium endemic countries affects the HBV prevalence [2,3]. Each year, around 1,800 HBV infections, 79% of which are chronic, are reported in the Netherlands [4]. Chronic HBV infections cause 23% of all liver cancers in the Netherlands, and are an important problem in ethnic minority groups, such as the Turkish community [5,6]. While this community represents 8% of the total city population in Rotterdam, it accounts for 30% of reported chronic HBV infections [7]. Seventy percent of reported infections among Turks involve people aged between 16 and 40. In this age-category, the mean incidence of reported HBV infections is 122 per 100,000 Turkish-Dutch individuals, much higher than the 35 infections per 100,000 persons reported in the total population of Rotterdam (Municipal Public Health Services Rotterdam-Rijnmond (MPHS), unpublished data, Rotterdam, 2007). However, these figures underestimate the population-prevalence: many chronic HBV-patients do not have disease symptoms, and are not reported. Population-based studies indicate a prevalence of chronic HBV of 0.2% in the general Dutch population, and a prevalence of 2.6 - 4.8% in the Turkish-Dutch population [6,8-10].

Most reported patients with chronic HBV have acquired HBV from their mother at birth [4]. Later in life, however, transmission is mainly through sexual contact [11].

HBV control now focuses both on pregnancy screening and on vaccinating risk groups, such as newborns from HBV-infected mothers, children with parent(s) from an HBV-endemic area, and people with high-risk behaviour [11]. These programmes however, have not contributed to the health of the general adult Turkish-Dutch population, leaving a substantial part of this population both undetected and unprotected regarding HBV.

In the past decade, treatment possibilities of chronic HBV have improved [12]. In order to detect individuals eligible for treatment and to prevent horizontal transmission in sexually (pre-)active individuals, screening for HBV should be promoted specifically in the Turkish-Dutch population. Public health interventions should target those who are least likely to participate in screening. Studies in Asian-American migrant groups, have shown that a lower screening rate is related to demographic factors - such as younger age, lower level of education, poor language proficiency, lower socio-economic status, and not having a health insurance - and lower levels of knowledge and awareness regarding HBV [13-22].

This study is a first step in developing an intervention aimed at the promotion of HBV-screening in the Turkish-Dutch population in Rotterdam. In order to target this intervention adequately, the current study aimed to determine 1) the levels of awareness and knowledge regarding HBV, comparing these in tested and non-tested members of the Turkish-Dutch population in Rotterdam; 2) the self-reported HBV test status in this population, and how this is related to demographic characteristics. In the next phase, we will determine causal relationships between behavioural and cultural determinants and HBV-screening behaviour.

## Methods

A sample of 1176 inhabitants of Rotterdam was drawn from the municipal administration. Included were people born in Turkey (first-generation migrants (FGM)); and people born in the Netherlands, with FGM parents (second-generation migrants (SGM)). Stratification was done on the basis of gender, migrant generation, and 5-year age group to ensure a minimum number of participants in each stratum. In order to over-sample strata in which a lower response was anticipated, we used response percentages reported for a health survey in the same population [23].

The questionnaires were translated and back-translated by two Turkish-Dutch translators. Inconsistencies in the translation and different understandings of concepts were discussed until consensus was reached between the translators.

One week after an announcement from the MPHS, asking for participation in the survey, FGM received a letter and questionnaire in both Turkish and Dutch language. SGM received the letter and questionnaire in Dutch only. Turkish-spoken support in filling out the questionnaire was available through telephone contact. Reminder packages consisting of a letter and questionnaire were sent to those who had not responded within three weeks. One week thereafter, all non-responders registered in the public telephone register were called and asked to respond. Furthermore, we stimulated participation by giving interviews on local radio stations, and by organising a lottery of gift vouchers among respondents.

Data-collection took place from March to May 2008. The Medical Ethical Review Board of Erasmus MC, University Medical Center Rotterdam, approved this study.

Development of the questionnaire (Additional file 1) was guided by focus group discussions in the Turkish community in Rotterdam [24]. The questionnaire contained the following sections:

Socio-demographic factors were sex, age, first- or second-generation migrant status, marital status, country of birth of partner, level of education (low-medium-high), socio-economic status (SES) of the residential area (categorized in low-mid/high SES), income situation, type of health insurance and Dutch language proficiency and use. Questions on the history of Hepatitis B testing and vaccination included test results and the persons' experience with HBV in family and friends. Awareness was measured through four separate items about the frequency of having thought about HBV in the past year. Answers could be given on a three-point scale 'never (1) - sometimes (2) - often (3)'. Knowledge was measured by ten statements, on which respondents could answer true, not true, or I don't know. Six statements on transmission and consequences were derived from a questionnaire by Taylor et al. [15]. We also formulated two statements on prevention. As focus group discussions in this population had indicated confusion in knowledge about hepatitis A and B, the last two statements assessed this issue. The total individual knowledge score could range from 0-10.

We also measured social-cognitive and socio-cultural determinants of hepatitis B screening but these will be described in a separate paper.

Because we used stratification in our sampling, we weighted all demographic characteristics, test rates, and awareness and knowledge scores by sex, age group and migrant generation, to be representative for the 16 to 40 year old Turkish-Dutch population in Rotterdam as per 1 January 2008. We compared tested and non-tested individuals regarding awareness and knowledge by using the chi-squared statistic. For this analysis, we dichotomized the awareness scores into low level (never thought about [the item] in the past year) versus high level (sometimes or often thought about [the item] in the past year), and we dichotomized the knowledge scores into 1 (correct answer) versus 0 (incorrect answer/'don't know'). We then used logistic regression analysis, adjusted for the stratification variables sex, age and migrant generation, to summarize the independent associations of demographic characteristics with having been tested for HBV. In the multivariate logistic regression analysis, we used stepwise backward selection of the variables which univariately showed a p-value ≤ 0.15. Finally, a second regression model was built which included the levels of awareness and knowledge, next to the demographic variables. In this multivariate analysis, we summarized the overall awareness into low (never thought about any of the awareness items in the past 12 months) versus high (at least thought about one of the four awareness-items in the past 12 months). We summarized the overall knowledge into low (0-5 correct answers) versus high (> 5 correct answers).

## Results

The response rate was 30.2% (n = 355). In the past 12 months, 27% of the respondents (n = 97) had thought at least once about one or more of the four awareness items (Table 1). This overall level of awareness differed between tested and non-tested individuals: 42% of tested people had some awareness, in contrast with 24% in the non-tested group (p < .01). Proportionately more tested than non-tested people had thought about the various awareness questions, except for the question about the risk of a family member contracting HBV. Both groups had hardly thought about this risk (10% and 9%, p = .8).

**Table 1:** Level of awareness regarding Hepatitis B in the Turkish-Dutch population in Rotterdam (weighted analysis)

	total	tested	non-tested	
	n = 355	n = 52	n = 303	
	%	%	%	p-value
Persons who in the past 12 months have at least sometimes thought about				
the disease Hepatitis B	20	32	18	0.01
the personal risk in contracting Hepatitis B	17	29	15	< 0.01
the risk of a family member contracting Hepatitis B	9	10	9	0.8
having a test for Hepatitis B	13	24	11	0.02
Overall (% of respondents who have thought about at least one of the four items)	27	42	24	< 0.01

The first three knowledge items focussed on HBV transmission. The average proportion of respondents who answered these items correctly was 54% (Table 2). For the three items, which tested knowledge about the consequences of HBV, the average proportion was 35%; for the two items about the prevention of HBV it was 68%. On average, 33% of respondents gave correct answers to the two statements about the difference between hepatitis A and B. Overall, the level of knowledge was higher in people who had been tested. The exceptions were the knowledge about the transmission of HBV during childbirth, which was about equally known by tested and non-tested (62% vs. 53%, p = .22), and the prevention of HBV by screening, which was known by 84% in both groups (p = .8). The level of knowledge about hepatitis A and B vaccination was equal in both groups (64% vs. 52%, p = .14). Fifty eight percent of the respondents answered less than six items correctly. There were no significant differences between men and women regarding awareness and knowledge, although men tended to be less aware of HBV (p = .08). Awareness in FGM and SGM was equally low; the awareness of SGM men younger than 30 being the poorest. FGM appeared to have less knowledge than SGM (p = .06), with SGM women (21-30 year) having the highest level of knowledge. There were no significant differences in awareness and knowledge between the age groups, although the age group 26-30 years had slightly more knowledge with 55% scoring 6 or more of the items correctly, compared to 42% in the total of all age groups.

**Table 2:** Proportion of people with correct knowledge about Hepatitis B among the tested and non-tested Turkish-Dutch population in Rotterdam (weighted analysis)

	total	tested	non-tested	
	n = 355	n = 52	n = 303	
	%	%	%	p-value
Transmission				
Hepatitis B cannot be spread by someone that looks and feels healthy.	54	68	51	0.03
Hepatitis B can be spread during childbirth.	54	62	53	0.22
Hepatitis B can be spread during sexual intercourse.	53	67	50	0.02
Average proportion for transmission	54	66	51	0.02
Consequences of HBV				
People with Hepatitis B can be infected for life.	44	69	39	< 0.001
Hepatitis B can cause liver cancer.	25	43	22	< 0.001
People can die from Hepatitis B.	36	53	34	< 0.01
Average proportion for consequences	35	55	32	< 0.001
Prevention				
Infection with Hepatitis B can not be prevented.	52	75	48	< 0.001
By being tested for Hepatitis B, one can find out whether one is infected.	84	84	84	0.8
Average proportion for prevention	68	79	66	0.001
Difference Hepatitis A (HAV) and HBV				
Hepatitis A and B are transferred from one person to the other in the same way.	13	20	12	0.15
Vaccination for both Hepatitis A and B are available.	54	64	52	0.14
Average proportion for difference HAV and HBV	33	41	32	0.16
Percentage of respondents with a high score (i.e. 6 or more correct answers)	42	71	37	< 0.001

Univariate logistic regression analysis of the level of awareness and knowledge with regard to having been tested for HBV, showed that relatively more people with some awareness (i.e. those who had at least thought about one of the four awareness-items in the past year) had been tested (OR 2.7 (1.5-4.8, p < .001) than people who had no awareness at all. It also showed that relatively more people with a knowledge-score of at least 6 out of 10, had been tested (OR 3.6 (2.0-6.6), p < .001) than people with a lower knowledge-score.

Although in the weighted analysis 52 people (14.7%) reported having been tested for HBV, only 42 of them reported the test results. In 86% (36/42) no antibodies against HBV had been detected in the blood. Four people reported that the screening had shown the presence of anti-HBc (antibodies to HBV core antigen), indicating infection with the virus in the past resulting in immunity. Four out of the 42 people (9.5%) reported to be carriers of HBV. Regarding vaccination, eleven respondents (11/355, 3%) were sure to have received full vaccination against HBV (i.e. 3 shots); while another 41 (12%) had not received the full series or were not sure about the completeness of the vaccination series. The majority of respondents had not been vaccinated (37%) or did not know whether they had been vaccinated (48%).

Univariate analysis showed that proportionately more respondents who knew family members or friends with HBV had been tested than those who did not (OR 3.4 (1.7 - 6.7), p < .001) (Table 3). In the multivariate model which included the factors gender, age, migrant generation, marital status and knowing someone with HBV, this latter factor remained significantly related to having been tested (OR 3.4 (1.7 - 6.7), p < .001). When including the levels of awareness and knowledge in the multivariate analysis, being married (OR 2.4 (1.1 - 5.2), p < .05), and higher levels of awareness (OR 2.3 (1.3 - 4.3, p < .01) and knowledge (OR 3.8 (2.0-7.1, p < .001) remained significantly related to having been tested. In this second model, knowing someone with HBV was borderline significantly related to having been tested for HBV (p = .06).

**Table 3:** Hepatitis B test-rates related to demographic factors in the Turkish-Dutch population in Rotterdam, the Netherlands (n = 355)

			total	Tested^c^	crude OR (univariate)	p-value	adjusted OR (multivariate)^e^	p-value
			n = 355	n = 52				
**Total**			100%	14.7%				
**Sex**		female	54%	15%	1.1 (0.6-1.8)	0.8	1.1 (0.6-2.0)	0.7
		male	46%	14%	ref			
**Age group**						0.13		0.1 (overall)
		16-20	19%	11%	ref			
		21-25	16%	12%	1.2 (0.4-3.7)	0.7	1.5 (0.5-5.0)	0.5
		26-30	20%	21%	1.9 (0.7-5.1)	0.2	2.3 (0.8-6.7)	0.1
		31-35	21%	11%	1.6 (0.6-4.4)	0.3	1.7 (0.6-4.8)	0.36
		36-40	25%	21%	2.9 (1.2-7.3)	0.02	3.4 (1.3-7.4)	0.02
**Migrant generation^a^**		1st generation	49%	16%	ref			
		2nd generation	51%	13%	1.2 (0.7-2.1)	0.5	1.4 (0.8-2.5)	0.3
**Marital status**		married/living with partner	59%	19%	1.8 (0.9-3.8)^d^	0.10		
		previously/never married	41%	9%	ref			
**Country of birth of partner^b^**		not/low endemic	29%	19%	1.3 (0.6-2.7)^d^	0.6		
		high endemic	71%	17%	ref			
**HBV in family or friends**		yes	15%	36%	**3.4 (1.7-6.7)**^d^	**< 0.001**	**3.4 (1.6-6.7)**	**< 0.001**
		no	85%	11%	ref			
**Educational level**						0.23		
		low	32%	14%	1.1 (0.5-2.4)^d^	0.8		
		medium	42%	13%	ref			
		high	26%	16%	1.9 (0.9-3.8)^d^	0.08		
**SES suburb**		low SES suburb	63%	15%	1.3 (0.7-2.3)^d^	0.4		
		medium/high SES suburb	37%	15%	ref			
**Income situation**						0.3		
		paid job	66%	13%	ref			
		social security	8%	19%	1.0 (0.4-3.1)^d^	0.9		
		fulltime housework	13%	26%	2.4 (1.0-6.0)^d^	0.05		
		student	14%	10%	0.9 (0.2-3.2)^d^	0.9		
**Health insurance**		basic health insurance	40%	13%	ref			
		basic + supplementary	60%	16%	1.4 (0.7-2.7)^d^	0.3		
**Dutch language orientation (proficiency and use)**		low level	47%	17%	ref			
		high level	53%	13%	1.3 (0.7-2.5)^d^	0.5		

^a ^1st generation migrant i.e. person born in Turkey. 2nd generation migrant i.e. person born in the Netherlands, with at least one parent born abroad.

^b ^n = 233

^c ^weighted analysis to correct for the stratification variables sex, age group and migrant generation

^d ^adjusted for the stratification variables sex, age group and migrant generation

^e ^the stratification variables sex, age group and migrant generation were retained in the final model

## Discussion and Conclusions

This study shows that the level of awareness regarding HBV in the Turkish-Dutch population is low. While HBV is a serious health problem in this community, over 70% of respondents have never thought about it in the past year. Knowledge about transmission and prevention of HBV is moderate, while there is especially little knowledge about the serious consequences of HBV. In this study, low HBV test- and vaccination rates are reported (15% and 3%, respectively). Test rates are even lower in people who are not married, or have lower levels of awareness and knowledge.

This study is the first research into awareness and knowledge regarding HBV and HBV-test rates in the Turkish community in the Netherlands, but it also has some limitations. Firstly, although we tried to stimulate response in various ways, the response rate was rather low (30.2%). This may be an indication of a lack of interest for the subject of hepatitis B which may jeopardize future participation in the intervention. The low response rate may also cause selection bias. Non-response analysis shows that non-respondents differed from respondents only with regard to gender (proportion female was 44% among non-respondents versus 54% among respondents), and not to age, migration generation and socio-economic status. Furthermore, the reported percentage of HBV carriers in our study is 9.5%, while we expected this to be between 2.6 - 4.8% [6,8-10]. This indicates that persons affected by HBV might have been more willing to respond, and that actual levels of awareness and knowledge in the population might be even lower than presented in this study. Secondly, information bias might have occurred, as in our questionnaire we gave away some information about testing and vaccination. This may have resulted in higher knowledge-scores on the prevention items. Thirdly, self-reports of screening and vaccination may be affected by inaccurate recall or desirability bias. Fourthly, we cannot assume causality between the factors on the one hand and having been tested on the other, because of the cross-sectional research design. Last, it is not likely that multiple testing has biased the conclusions as we found a considerable number of significant differences between the groups, with p-values below 0.001.

We found low levels of knowledge regarding the consequences of HBV. Studies in Asian migrants in the USA found higher levels of HBV knowledge [25]. One possible explanation is that HBV is an even more prevalent health problem in Asian populations, than in the Turkish population. Another is that knowledge may have been improved by health education activities in the country of origin or in the host country [19]. As far as we know, this has not been the case for Turkish migrants coming to the Netherlands. These health education activities may also have influenced the test rates amongst Asian migrants in the USA (range 8 - 68%) [15,16,18,19,25-27], which were considerably higher than the test rate we found amongst Turkish Dutch (14.7%).

Since 1989, national policy has prescribed HBV-screening for pregnant women. In our study, about 25% of the married females reported to have been tested. This proportion appears to be low in view of the fact that, based on demographic trend information in migrant women in the Netherlands, we estimate that in reality about 50% of all married females may have been tested during pregnancy [28,29]. This would result in a total test rate of 22%, instead of the reported 15%. The women who underreported screening, are likely women who tested negative for HBV and are susceptible to the virus. As the aim of our intervention is both detecting HBV and protecting against HBV, we also target our intervention to these women in order to provide them with adequate preventive measures. Last, it is likely that screened women who appeared to be carriers are aware of having been tested, and therefore the underreporting does not affect the carrier rate.

Current screening guidelines also include source and contact tracing, which means that invitations for HBV-screening are extended to plausible source(s) and contacts of a notified HBV-carrier. This may explain the results of the regression analyses, which showed that the factors 'being married' and 'knowing a family member or friend with HBV' were (borderline) related to having been screened. The first time most of the Turkish-Dutch women will be tested for HBV is during pregnancy; which seldom occurs before marriage [30]. The fact that knowing someone with HBV is related to previous HBV-testing has been shown in other studies [16] as well as in our own. This may be due to HBV-affected family members or friends who are prompted to be tested themselves, or to the source and contact tracing.

Several studies found an association between higher age and having been tested [19,31]. Although in our study we found a tendency that older people were more often tested, this relation was not significant. While other studies also found that the level of education, language proficiency, and level of health insurance were associated with previous testing, our findings did not confirm this. Almost everyone in our study had a health insurance, and this factor was not associated with having been screened. It is suggested that in areas with high levels of health-care coverage, the influence of being insured has less effect on actually being screened [26]. This might also be valid for the level of education, which was high in our study.

This study shows that the Turkish population in Rotterdam has low levels of awareness and knowledge regarding HBV, and low rates of HBV-testing and -vaccination. While the national HBV-screening policy in the Netherlands covers mainly pregnant women and their contacts, the risk of HBV is present in the whole Turkish-Dutch population. In order to prevent HBV-transmission in adults, it would be useful to test people before they become sexually active. The findings in the present study show that the development of a health promotion intervention regarding HBV should raise awareness about the risk of HBV in this population, and particularly address the serious consequences of HBV.

## Competing interests

The authors declare that they have no competing interests.

## Authors' contributions

OZ and JHR and made substantial contributions to the conception and design of this study and revised the manuscript critically. YV organised the survey, analysed the data, and drafted the manuscript. YV and HV were involved in data-interpretation and in revising the manuscript. All authors read and approved the final manuscript.

## Pre-publication history

The pre-publication history for this paper can be accessed here:

http://www.biomedcentral.com/1471-2458/10/512/prepub

## Supplementary Material

Additional file 1 **Questionnaire on hepatitis B, testing and vaccination in the Turkish community in Rotterdam**. Questionnaire on hepatitis B, testing and vaccination in the Turkish community in Rotterdam, including demographic items, questions on awareness and knowledge, and the history of testing and vaccination. Click here for file
