# Supplementary material for: Awareness, knowledge and self-reported test rates regarding Hepatitis B in Turkish-Dutch: a survey
Source: BMC Public Health. 2010 Aug 24;10:512. doi: 10.1186/1471-2458-10-512 (PMC2940913; doi:10.1186/1471-2458-10-512)
Supplement: Additional file 1 — Questionnaire on hepatitis B, testing and vaccination in the Turkish community in Rotterdam. Questionnaire on hepatitis B, testing and vaccination in the Turkish community in Rotterdam, including demographic items, questions on awareness and knowledge, and the history of testing and vaccination. [file 1471-2458-10-512-S1.DOC]

***Questionnaire Hepatitis B testing among Turkish Dutch***

***<introduction letter>***

***<instruction for filling out the questions>***

***Let’s start with some general questions. Please, check the box next to your preferred answer, or write it down in the available text boxes.***

1. Are you male or female?

□ Male

□ Female

1. What is your year of birth? □ □ □ □
2. Please, write down your postal code: □ □ □ □
3. In which country were you born?

□ the Netherlands → continue with question 6

□ Turkey

□ another country:

1. In which year did you start to live in the Netherlands? □ □ □ □
2. In which country was your father born?

□ the Netherlands

□ Turkey

□ another country:

1. In which country was your mother born?

□ the Netherlands

□ Turkey

□ another country:

1. What is your civil class?

□ married, registered partnership

□ living together

□ single (never been married) → continue with question 10

□ divorced

□ widowed

In this questionnaire, we use the term ‘partner’ which may be your wife or husband or a person you are in a steady relationship with.

1. In which country was your partner born?

□ the Netherlands

□ Turkey

□ another country:

1. What is the highest level of education you received? (Any education with a diploma or official certificate.) Please, choose the level of education that is closest to your level of education.

□ No education (did not finish primary education)

□ Primary education

□ Lower level vocational training

□ Medium level vocational training

□ Bachelor degree

□ Master’s degree

□ other education:

1. Which situation is most like yours? You may check more than one box.

| □ | I have a paid job |
| --- | --- |
| □ | I receive a pension |
| □ | I receive payment from social security |
| □ | I am a fulltime household worker |
| □ | I study |

1. How are you insured against health costs?

□ basic insurance

□ basic insurance plus additional insurance

□ no health care insurance

***Hepatitis B is a disease, which is caused by a virus. We would now like to ask you some questions about your experience with hepatitis B.***

1. Do you know persons living with hepatitis B?

□ yes, it occurs in my family

□ yes, it occurs in my circle of friends/acquaintances

□ yes, it occurs in my family and in my circle of friends/acquaintances

□ no

1. Please, indicate below whether you think the following statements regarding hepatitis B are true or false. Check the box next to your preferred answer.

**Attention!** Please, do not guess the answer. If you do not know the answer, you may indicate ‘don’t know’.

|  |  | **true / false / don’t know** |
| --- | --- | --- |
| 14a | If someone has hepatitis B, but looks and feels healthy, this person cannot infect others with the virus. | □  □   □ |
| 14b | During a delivery, a mother may infect her child with hepatitis B virus. | □  □   □ |
| 14c | Hepatitis B may be transmitted by sexual intercourse with a person who has hepatitis B. | □  □   □ |
| 14d | People may carry the hepatitis B virus life long, and infect others with it. | □  □   □ |
| 14e | Hepatitis B may cause liver cancer. | □  □   □ |
| 14f | Infection with Hepatitis B cannot be prevented. | □  □   □ |
| 14g | Hepatitis B can cause death. | □  □   □ |
| 14h | By getting tested for hepatitis B, one may find out whether he/she is infected. | □  □   □ |
| 14i | Hepatitis A and hepatitis B are transmitted between humans in the same way. | □  □   □ |
| 14j | Vaccination for both hepatitis A and hepatitis B is available. | □  □   □ |

**At your GP, the MPHS or in a hospital a test for hepatitis B may be done by taking some blood from the arm. In the blood it may show whether someone has been infected with hepatitis B virus.**

1. Have you ever been tested for hepatitis B?

□ yes, I have been tested for hepatitis B

□ no, I have not been tested for hepatitis B → continue with question 22

□ I do not know whether I have been tested for hepatitis B → continue with question 22

1. What was the result of the test?

□ I had not been in contact with the hepatitis B virus (not infected)

□ I had had a hepatitis B virus infection, but the virus was cleared

□ I carry the hepatitis B virus

□ I don’t recall the result of the test

1. In which year were you tested for hepatitis B? (If you do not recall, just estimate the year.)

□ □ □ □

1. In which country were you tested?

□ the Netherlands

□ Turkey

□ another country:

1. Why were you tested for hepatitis B?

□ I myself wanted to know whether I had hepatitis B → continue with question 20

□ the MPHS asked me to come forward for a test → continue with question 21

□ another reason:

**→ please, fill out either question 20 and/or 21**

1. Why did you decide to have test? Please, write your answer in the text box here below.

**→ continue with question 22**

1. Do you know why the MPHS asked you to come for a test? Please, write your answer in the text box here below.
2. Are you vaccinated for hepatitis A and/or B? If you are not sure which vaccination you received, please read the information in the box on the next page.

□ Yes, I am vaccinated for both hepatitis A and hepatitis B → go to question 23

□ I am just vaccinated for hepatitis A, not for hepatitis B → go to question 25

□ I am just vaccinated for hepatitis B, not for hepatitis A → go to question 23

□ I am not vaccinated for either hepatitis A or B → go to question 25

□ I am not sure whether I am vaccinated for hepatitis A and/or B → go to question 25

**Information Hepatitis B vaccination**

During a test for hepatitis B, blood is taken from your arm. During a vaccination something is injected into your arm. Vaccination for hepatitis B comprises 3 injections in the upper arm, so that the body may produce antibodies against the hepatitis B virus, and so built up immunity. When the virus tries to enter the body after vaccination, the antibodies will kill the virus, and you will not become ill due to it.

In Turkey, this vaccination is recommended for children since 1998. In the Netherlands, this vaccination is included in the National Vaccination Programme that is conducted at the child clinics. All children with at least one parent born in Turkey receive this series of vaccination.

All persons born before 2003 may obtain vaccination, when there is a risk for contracting hepatitis B. This may be done by the GP or at the MPHS. After vaccination, someone cannot be infected with the virus anymore.

There are other forms of hepatitis, such as hepatitis A en C. All kinds of hepatitis are contagious. Vaccinations for hepatitis A are not included in the National Vaccination Programme. This vaccination includes two injections, which are often given before someone travels to Turkey.

***Attention! The following questions only regard hepatitis B vaccination.***

1. Are you fully vaccinated for hepatitis B, meaning that you received 3 injections in your upper arm? *(You may want to look this up in the so called ‘yellow booklet’.)*

□ Yes, I am fully vaccinated for hepatitis B

□ I am not sure whether I have been fully vaccinated for hepatitis B

□ I am not fully vaccinated for hepatitis B

1. In which year were you vaccinated for hepatitis B? (If you do not recall, just estimate the year.)

□ □ □ □

## We would now like to ask your opinion about testing for hepatitis B.

1. Have you thought about the following issues in the past 12 months? Please, check a box on each of the four lines here below.

| How often have you thought about the disease hepatitis B in the past 12 months? | never – sometimes – often   □  □  □ |
| --- | --- |
| How often have you thought about the personal risk in contracting hepatitis B in the past 12 months? | never – sometimes – often   □  □  □ |
| How often have you thought about the risk of a family member contracting hepatitis B in the past 12 months? | never – sometimes – often   □  □  □ |
| How often have you thought about having a test for hepatitis B in the past 12 months? | never – sometimes – often   □  □  □ |

1. How much do you agree or disagree with the statements here below? Please, check the box of your preferred answer on each of the lines.

|  | **Having a hepatitis B test…** | **totally disagree** | **disagree** | **neutral** | **agree** | **totally**  **agree** |
| --- | --- | --- | --- | --- | --- | --- |
| 26a | is good for my health | □ | □ | □ | □ | □ |
| 26b | gives me clarity about whether I have hepatitis B or not | □ | □ | □ | □ | □ |
| 26c | helps to prevent that I infect others | □ | □ | □ | □ | □ |
| 26d | is expensive | □ | □ | □ | □ | □ |
| 26e | costs a lot of time | □ | □ | □ | □ | □ |
| 26f | is not the most important thing I can do for my health right now | □ | □ | □ | □ | □ |
| 26g | is unnecessary when I don’t have any physical complaints | □ | □ | □ | □ | □ |

## We now would like to know how you think about the opinion of people near to you.

1. Do people near to you (neighbours, family, friends) feel it is important to care for good health?

□ very important

□ important

□ not important

□ not important at all

□ I don’t know

1. Do people near to you (neighbours, family, friends) feel it is important to encourage other people to live healthy?

□ very important

□ important

□ not important

□ not important at all

□ I don’t know

1. How much do you agree or disagree with the following statements?

29.a. My partner thinks it is important □ totally disagree

that I be tested for hepatitis B. □ disagree

□ don’t agree, don’t disagree

□ agree

□ totally agree

□ I don’t have a partner

29b. My parents think it is important □ totally disagree

that I be tested for hepatitis B. □ disagree

□ don’t agree, don’t disagree

□ agree

□ totally agree

□ I don’t have parents (anymore)

29c. My friends think it is important □ totally disagree

that I be tested for hepatitis B. □ disagree

□ don’t agree, don’t disagree

□ agree

□ totally agree

□ no friends

1. How much do you agree or disagree with the following statements?

30a. If I would be tested for hepatitis B, my partner □ totally disagree

would support me in doing so. □ disagree

□ don’t agree, don’t disagree

□ agree

□ totally agree

□ I don’t have a partner

30b. If I would be tested for hepatitis B, my parents □ totally disagree

would support me in doing so. □ disagree

□ don’t agree, don’t disagree

□ agree

□ totally agree

□ I don’t have parents (anymore)

30c. If I would be tested for hepatitis B, my friends □ totally disagree

would support me in doing so. □ disagree

□ don’t agree, don’t disagree

□ agree

□ totally agree

□ no friends

1. How important is the opinion of these persons for you?

|  |  | **very important** | **important** | **not**  **important** | **not important at all** |
| --- | --- | --- | --- | --- | --- |
| 31a | the opinion of your partner | □ | □ | □ | □ |
| 31b | the opinion of your parents | □ | □ | □ | □ |
| 31c | the opinion of friends | □ | □ | □ | □ |

1. If the test result would show you are infected with hepatitis B virus, how would you feel?

***Please, check a box on each of the five lines here below..***

|  |  | **definitely not** | **probably not** | **probably** | **definitely** |
| --- | --- | --- | --- | --- | --- |
| 32a | I would feel embarrassed. | □ | □ | □ | □ |
| 32b | I would feel ashamed. | □ | □ | □ | □ |
| 32c | I would feel disappointed in myself. | □ | □ | □ | □ |
| 32d | I would feel guilty. | □ | □ | □ | □ |
| 32e | I would feel scared. | □ | □ | □ | □ |

1. If the test result would show you are infected with hepatitis B virus, and others would come to know, do you think:

|  |  | **definitely not** | **probably not** | **probably** | **definitely** |
| --- | --- | --- | --- | --- | --- |
| 33a | they would avoid you? | □ | □ | □ | □ |
| 33b | they would think you were unclean? | □ | □ | □ | □ |
| 33c | they would still be friends with you? | □ | □ | □ | □ |
| 33d | they would less respect you? | □ | □ | □ | □ |
| 33e | they would feel uncomfortable around you? | □ | □ | □ | □ |

1. In your community, is it important what other people think about you?

□ very important

□ important

□ not important

□ not important at all

□ I don’t know

1. Imagine you would want to be tested for hepatitis B, or you have been tested already. How easy or difficult is/was it for you to perform the following tasks? This question is not about getting tested or not, but how you would feel if you had decided to be tested.

*Please, check the box below your preferred answer. The last column is only to be checked when you do not have a partner and/or parents (anymore).*

|  | ***How would it be for you to*** | **very difficult** | **difficult** | **neither difficult nor easy** | **easy** | **very easy** | **n.a.** |
| --- | --- | --- | --- | --- | --- | --- | --- |
| 35a | tell your parents you want to be tested. | □ | □ | □ | □ | □ | □ |
| 35b | tell your partner you want to be tested. | □ | □ | □ | □ | □ | □ |
| 35c | make an appointment with the MPHS to get tested. | □ | □ | □ | □ | □ |  |
| 35d | get tested when your parents would not agree with it. | □ | □ | □ | □ | □ | □ |
| 35e | get tested when your partner would not agree with it. | □ | □ | □ | □ | □ | □ |
| 35f | share the result of the test with your parents, when it shows that you have hepatitis B. | □ | □ | □ | □ | □ | □ |
| 35g | share the result of the test with your partner, when it shows that you have hepatitis B. | □ | □ | □ | □ | □ | □ |
| 35h | discuss your personal issues with a Dutch doctor. | □ | □ | □ | □ | □ |  |
| 35i | discuss your personal issues with a Turkish doctor. | □ | □ | □ | □ | □ |  |

1. How serious do you rate the diseases mentioned below?

|  |  | **not serious**  **at all** | **not serious** | **serious** | **very serious** |
| --- | --- | --- | --- | --- | --- |
| 36a | hepatitis B | □ | □ | □ | □ |
| 35b | flu | □ | □ | □ | □ |
| 36c | AIDS | □ | □ | □ | □ |
| 36d | cancer | □ | □ | □ | □ |
| 36e | hearth attack | □ | □ | □ | □ |

1. Please, indicate here below how likely it is you contract hepatitis B.

|  | ***How likely is it that you*** | **very unlikely** | **unlikely** | **not unlikely,**  **not likely** | **likely** | **very likely** |
| --- | --- | --- | --- | --- | --- | --- |
| a. | … already contracted hepatitis B? | □ | □ | □ | □ | □ |
| b. | … will contract hepatitis B in the next 5 year? | □ | □ | □ | □ | □ |
| c. | … will infect someone with hepatitis B in the next 5 year? | □ | □ | □ | □ | □ |

***We now like to ask some questions about your opinion about the Turkish and Dutch health care.***

1. How do you feel about the statements here below?

|  |  | **totally disagree** | **disagree** | **don’t agree, don’t disagree** | **agree** | **totally**  **agree** |
| --- | --- | --- | --- | --- | --- | --- |
| 38a | The costs of a test in the Netherlands will be higher than those in Turkey. | □ | □ | □ | □ | □ |
| 38b | In the Netherlands, it takes a long time to get an appointment with a doctor. | □ | □ | □ | □ | □ |
| 38c | I am satisfied with the Dutch health care. | □ | □ | □ | □ | □ |
| 38d | It is difficult for me to communicate with a Dutch doctor, because of my language difficulties. | □ | □ | □ | □ | □ |
| 38e | Doctors in Turkey have more experience regarding hepatitis B than doctors in the Netherlands. | □ | □ | □ | □ | □ |

1. In your community, are there outspoken ideas about the quality of health care in the Netherlands?

□ yes, people think about it in a positive way

□ yes, people think about it in a negative way

□ no, there are no outspoken ideas about the quality of Dutch health care

□ I don’t know

1. Imagine you would receive an invitation from the MPHS to get tested for hepatitis B within 3 months. Would you intend to get tested?

□ definitely

□ probably

□ maybe yes, maybe no

□ probably not

□ definitely not

1. What would be the most important reason for you to get tested? Please, check only one box.

□ I would do that, so I can be treated for it, if necessary.

□ I would do that, so I can take precautions not to infect others.

□ I feel these two reasons for testing are evenly important.

□ I feel these two reasons are not important.

□ I would be tested for another reason, which is:

***We now want to ask you some questions about family relationships.***

1. How do you feel about the following statements?

|  |  | **totally disagree** | **disagree** | **don’t agree, don’t disagree** | **agree** | **totally**  **agree** |
| --- | --- | --- | --- | --- | --- | --- |
| 42a | Children should care for there sick parents. | □ | □ | □ | □ | □ |
| 42b | When you are in trouble, your family should stand beside you. | □ | □ | □ | □ | □ |
| 42c | I rather discuss problems with family members than with friends. | □ | □ | □ | □ | □ |
| 42d | Family members should always be counted upon. | □ | □ | □ | □ | □ |
| 42e | Family members should always be ready to help one each other, even though they don’t like each other. | □ | □ | □ | □ | □ |
| 42f | I trust in my friends more than I trust in my family. | □ | □ | □ | □ | □ |
| 42g | My family members have close connections. | □ | □ | □ | □ | □ |
| 42h | We keep one another informed about important happenings in the family. | □ | □ | □ | □ | □ |

1. In your community, is the stake of one person or of that of the group more important?

□ the stake of one person is more important

□ the stake of the group is more important

□ both stakes are important

□ I don’t know

***Hepatitis B is a disease that is transmittable from mother to child, by blood contact and by sexual contact. We would like to know your opinions regarding sexuality.***

1. In your community, is it common to talk openly about sexuality?

|  |  | **yes** | **no** | **I don’t know** |
| --- | --- | --- | --- | --- |
| 44a | with my family | □ | □ | □ |
| 44b | with my friends | □ | □ | □ |
| 44c | with acquaintances | □ | □ | □ |

1. What do you think about the following statements?

|  |  | **totally disagree** | **disagree** | **don’t agree, don’t disagree** | **agree** | **totally**  **agree** |
| --- | --- | --- | --- | --- | --- | --- |
| 45a | In the Netherlands, men and women get along too easy (too liberal). | □ | □ | □ | □ | □ |
| 45b | In the Netherlands, people discuss sexuality too upright. | □ | □ | □ | □ | □ |
| 45c | To me, hepatitis B testing has to do with sexuality. | □ | □ | □ | □ | □ |
| 45d | If I get tested, others will think I had unsafe sex. | □ | □ | □ | □ | □ |
| 45e | If I get tested, others will think I had extra-marital sexual intercourse. | □ | □ | □ | □ | □ |

***In Rotterdam many cultural backgrounds are represented in its inhabitants. There are many ways of expressing these different ethnic backgrounds, such as Moroccans, Dutch, Turks, but also ‘Turkse Rotterdammers’, ‘Moroccan Dutch’ etc. The next questions are about your original ethnic background, how you feel about it, and how you deal with it.***

1. Which is your original ethnic group?

□ Turkish

□ Kurdish

□ Armenian

□ Arabic

□ Other:

1. How do you feel?

□ I feel completely Dutch

□ I feel predominantly Dutch

□ I feel as much Turkish as Dutch

□ I feel predominantly Turkish

□ I feel completely Turkish

□ I feel more like (another ethnic group):

1. Please, check the box to indicate whether you agree or disagree.

|  |  | **strongly disagree** | **somewhat disagree** | **somewhat agree** | **strongly**  **agree** |
| --- | --- | --- | --- | --- | --- |
| 48a | I spent time trying to find out more about my own ethnic group, such as its history, traditions and customs. | □ | □ | □ | □ |
| 48b | I am active in organisations or social groups that include mostly members of my own ethnic group. | □ | □ | □ | □ |
| 48c | I have a clear sense of my ethnic background and what this means to me. | □ | □ | □ | □ |
| 48d | I think a lot about how my life will be affected by my ethnic group membership. | □ | □ | □ | □ |
| 48e | I am happy that I am a member of the group I belong to. | □ | □ | □ | □ |
| 48f | I have a strong sense of belonging to my own ethnic group. | □ | □ | □ | □ |
| 48g | I understand pretty well what my ethnic group membership means to me. | □ | □ | □ | □ |
| 48h | In order to learn more about my ethnic background, I have often talked with other members of my ethnic group. | □ | □ | □ | □ |
| 48i | I have a lot of pride in my ethnic group. | □ | □ | □ | □ |
| 48j | I participate in cultural practices of my own group, such as special food, music or customs. | □ | □ | □ | □ |
| 48k | I feel a strong attachment towards my own ethnic group. | □ | □ | □ | □ |
| 48l | I feel good about my cultural or ethnic background. | □ | □ | □ | □ |

1. **Please, check the boxes to indicate whether you agree or disagree with the following statements.**.

|  |  | **totally disagree** | **disagree** | **don’t agree, don’t disagree** | **agree** | **totally**  **agree** |
| --- | --- | --- | --- | --- | --- | --- |
| 49a | I like meeting and getting to know people from ethnic groups, other than my own. | □ | □ | □ | □ | □ |
| 49b | I sometimes feel it would be better if different ethnic groups didn’t try to mix together. | □ | □ | □ | □ | □ |
| 49c | I often spend time with people from other ethnic groups. | □ | □ | □ | □ | □ |
| 49d | I don’t try to become friends with people from ethnic groups other than my own. | □ | □ | □ | □ | □ |
| 49e | I am involved in activities with people from other ethnic groups. | □ | □ | □ | □ | □ |
| 49f | I enjoy being around people from ethnic groups other than my own. | □ | □ | □ | □ | □ |

# *Research shows that faith or religion may influence how people view and deal with health. Therefore, we would like you to answer the next questions about religion.*

1. Are you religious?

□ yes

□ no → continue with question 54

1. To which religion do you consider to belong to?

□ Islam

□ Jewish

□ Christianity

□ Another religion: → continue with question 54

1. In your religious community, are there rules about:

|  |  | **yes** | **no** | **I don’t know** |
| --- | --- | --- | --- | --- |
| 52a | how to deal with disease | □ | □ | □ |
| 52b | how disease may be prevented | □ | □ | □ |
| 52c | responsibility for your own health | □ | □ | □ |
| 52d | responsibility for the health of others | □ | □ | □ |

1. *How do you feel about the next statements?*

|  |  | **totally disagree** | **disagree** | **don’t agree, don’t disagree** | **agree** | **totally**  **agree** |
| --- | --- | --- | --- | --- | --- | --- |
| 53a | Being religious is an important part of me. | □ | □ | □ | □ | □ |
| 53b | Being religious is something I think about often. | □ | □ | □ | □ | □ |
| 53c | I see myself as a real believer. | □ | □ | □ | □ | □ |
| 53d | When I am ill, I pray to God/Allah for healing. | □ | □ | □ | □ | □ |
| 53e | I trust that God/Allah cares for my health. | □ | □ | □ | □ | □ |
| 53f | As a believer, I feel that I should care well for my health. | □ | □ | □ | □ | □ |
| 53g | As a believer, I feel that I am responsible for the health of others. | □ | □ | □ | □ | □ |
| 53h | As a believer, I feel that I am obliged to do all I can for my health. | □ | □ | □ | □ | □ |

1. How do you feel about the next statements?

|  |  | **totally disagree** | **disagree** | **don’t agree, don’t disagree** | **agree** | **totally**  **agree** |
| --- | --- | --- | --- | --- | --- | --- |
| 54a | My good health is largely a matter of good fortune. | □ | □ | □ | □ | □ |
| 54b | If it's meant to be, I will stay healthy. | □ | □ | □ | □ | □ |
| 54c | No matter what I do, if I am going to get sick, I will get sick. | □ | □ | □ | □ | □ |
| 54d | Whatever happens to my health is God's will. | □ | □ | □ | □ | □ |
| 54e | I am in control of my health. | □ | □ | □ | □ | □ |
| 54f | The main thing which affects my health is what I myself do. | □ | □ | □ | □ | □ |
| 54g | If I take the right actions, I can stay healthy. | □ | □ | □ | □ | □ |
| 54h | If I take care of myself, I can avoid illness. | □ | □ | □ | □ | □ |

***Next follows a question about your Dutch language provision, and use of the media, such as news papers, TV, radio and the Internet.***

1. *Please, check the box below your preferred answer.*

**Attention!** In case you don’t have a partner and/or children you may check the ‘n.a.’ box as you like.

| Do you speak Dutch with your partner? | never – sometimes – often - always   □   □   □   □  | **na**  □ |
| --- | --- | --- |
| Do you speak Dutch with your children? | never – sometimes – often - always   □   □   □   □  | **na**  □ |
| Is it difficult for you to speak the Dutch language? | never – sometimes – often - always   □   □   □   □  |  |
| Is it difficult for you to read the Dutch language? | never – sometimes – often - always   □   □   □   □  |  |

1. Which newspapers or magazines do you read at least once a week?

□ Turkish

□ Dutch

□ Both

□ I never read newspapers or magazines

□ Other:

1. How often do you watch Dutch films, television or video programmes, or do you listen to Dutch radio broadcastings?

□ never

□ a few times per week

□ daily

1. How often do you watch Turkish films, television or video programmes, or do you listen to Turkish radio broadcastings?

□ never

□ a few times per week

□ daily

1. Do you have a satellite disc to receive foreign broadcastings?

□ yes

□ no

1. Do you have a personal computer at home?

□ Yes

□ No

□ I don’t know

1. Do you use the Internet?

□ Yes

□ No → go to question 64

1. How often do you use the Internet?

□ daily

□ a few times per week

□ once a week

□ less than once a week

1. Where do you use the Internet?

□ most often at home

□ most often at work

□ most often somewhere else:

1. Do other people living in your house use the Internet?

□ Yes, often at home

□ Yes, most often somewhere else:

□ No

□ I don’t know whether one of my housemates uses the Internet

1. The MPHS would like to provide you with information about hepatitis B. How would you like to receive this information?

*You may check a maximum of three boxes..*

□ in a group meeting with other people

□ by TV, in Turkish / Dutch language (please, cross the least preferred language)

□ by newspaper, in Turkish / Dutch language (please, cross the least preferred language)

□ by radio, in Turkish / Dutch language (please, cross the least preferred language)

□ by written information sent by post

□ during a meeting of a social organisation

□ during a visit to the hospital or GP

□ via internet, through the following websites: (please, note down website you often visit)

□ other channel:

1. The MPHS would like to invite you to get tested for hepatitis B. How would you like to be invited?

*You may check only one box here.*

□ in a group meeting with other people

□ by TV, in Turkish / Dutch language (please, cross the least preferred language)

□ by newspaper, in Turkish / Dutch language (please, cross the least preferred language)

□ by radio, in Turkish / Dutch language (please, cross the least preferred language)

□ by written information sent by post

□ during a meeting of a social organisation

□ during a visit to the hospital or GP

□ via internet, through the following websites: (please, note down website you often visit)

□ other channel:

***Finally, a last question on hepatitis C.***

1. Have you heard any information about hepatitis C in the past 6 months? You may check more than one box.

□ no → go the section ‘closing’

□ yes, in a group meeting

□ yes, from a health professional at the GP

□ yes, from family and friends

□ yes, from a different source:

1. What have you heard about hepatitis C ?

Closing

***Thank you so much for filling out this questionnaire!***

Would you like to join in a lottery of gift vouchers, as explained in the letter you received?

□ Yes □ No

Would you like to be informed about the results of this questionnaire?

□ Yes □ No

**By filling out this questionnaire, questions might have been raised about hepatitis B. It will take sometime before the health education materials will be published. Till that time, you might want to find out more about hepatitis B at www.hepatitis.nl.**
